# Reliability of the modified Rankin Scale in clinical practice of stroke units and rehabilitation wards

**DOI:** 10.3389/fneur.2023.1064642

**Published:** 2023-03-03

**Authors:** Natalia Pożarowszczyk, Iwona Kurkowska-Jastrzębska, Iwona Sarzyńska-Długosz, Maciej Nowak, Michał Karliński

**Affiliations:** ^1^2nd Department of Neurology, Institute of Psychiatry and Neurology, Sobieskiego, Warsaw, Poland; ^2^Neurological Rehabilitation Ward, Institute of Psychiatry and Neurology, Sobieskiego, Warsaw, Poland

**Keywords:** stroke, modified Rankin Scale, disability, reliability, consistency, outcome assessment

## Abstract

**Introduction:**

The Modified Rankin Scale (mRS) is the most common tool to quantify post-stroke disability in everyday practice and by certified raters in clinical trials. However, interobserver variability may affect reliability of retrospective observational studies, including clinical registries. Our aim was to assess real-life consistency between neurologists and physical and rehabilitation medicine physicians using mRS to rate post-stroke disability of patients transferred directly from stroke unit (SU) to rehabilitation ward (RW).

**Methods:**

This is a retrospective analysis of 132 consecutive acute stroke patients transferred from single tertiary SU to RW located in the same hospital in Poland. Patients were assessed by one rater from each department at the day of transfer. We distinguished between physicians previously certified in using mRS for clinical trials and not-certified physicians using mRS in everyday practice.

**Results:**

mRS at discharge from SU and on admission to RW was recorded for 105 of 132 patients. The overall agreement was 70.5% (kappa 0.55). Similar agreement was observed in the subset of 30 patients rated by certified physicians in both departments (70.0%, kappa 0.57) and in the subset of 61 patients rated by a pair of certified neurologist and not-certified rehabilitation physician (73.8%, kappa 0.58).

**Conclusions:**

Everyday consistency between raters from SU and RW in using mRS is modest as in previous validation studies. However, it may be considered sufficient for the purpose of observational studies or stroke registries. It emphasizes the need for easily accessible training in conventional mRS or implementation of specialized tools with predefined questions.

## Introduction

The modified Rankin Scale (mRS) has been the gold standard for measuring stroke outcome in clinical trials from many years (1–3). As a consequence, it has also become the standard in observational studies and is recommended for everyday use in clinical practice of stroke units and rehabilitation wards all over the world (1–3). This ordinal scale which grades patient's disability from 0 (no symptoms) to 6 (death) is able to capture the whole spectrum of functional states and poses intuitive simplicity (3–5). Despite those major advantages, the issue of significant interobserver variability may lead to end point misclassification and can affect results of clinical studies (6–8).

The attempts to improve the reliability of mRS assessment include the development structured interview, a formal simplified mRS questionnaire (smRSq) and introduction of certification video-based training provided by Glasgow University (*trainingcampus.net*) (9–12). However, most observational studies and multicenter registries are based on data collected in the course of everyday clinical practice. The assessments are made by attending physicians of different clinical backgrounds who are not always aware of all nuances of mRS and not as dedicated as investigators in clinical trials. This may rise an important question about the bias in retrospective analyses of observational data extracted from clinical records.

Our aim was to assess the real-life consistency between neurologists and physical and rehabilitation medicine (PRM) physicians using mRS to rate post-stroke disability in patients transferred directly from a stroke unit (SU) to a rehabilitation ward (RW).

## Methods

This is a retrospective analysis of all consecutive adult acute stroke patients treated in a single tertiary SU from January 2017 to December 2019 and were subsequently transferred to RW located in the same hospital in Warsaw, Poland.

Post-stroke disability was routinely measured using mRS (i) at the discharge from SU by the neurologist or neurologist trainee and (ii) on admission to RW by the specialist in PRM or PRM trainee. Some physicians working in the RW were also specialists in neurology. It is important to note that both mRS assessments were made at the day of transfer which makes significant changes in patients' functional state highly unlikely. The final analysis included only patients having mRS score stated directly in the last observation or discharge note from SU and stated directly in the initial observation or discharge note from RW. There were no exclusion criteria.

We additionally distinguished between (i) physicians who within the last 5 years preceding patients' assessment at least once completed formal mRS certification at *trainingcampus.net* for the purpose of clinical trials (certified mRS raters) and (ii) physicians who used the scale only as a part of everyday practice (non-certified mRS raters). The authors knew the mRS certification status of involved physicians, as they were either principal investigators (IK-J) or sub-investigators (MK, IS-D) in all stroke trials carried out in both departments within the last 10 years.

### Statistical analysis

**Figure 1:**
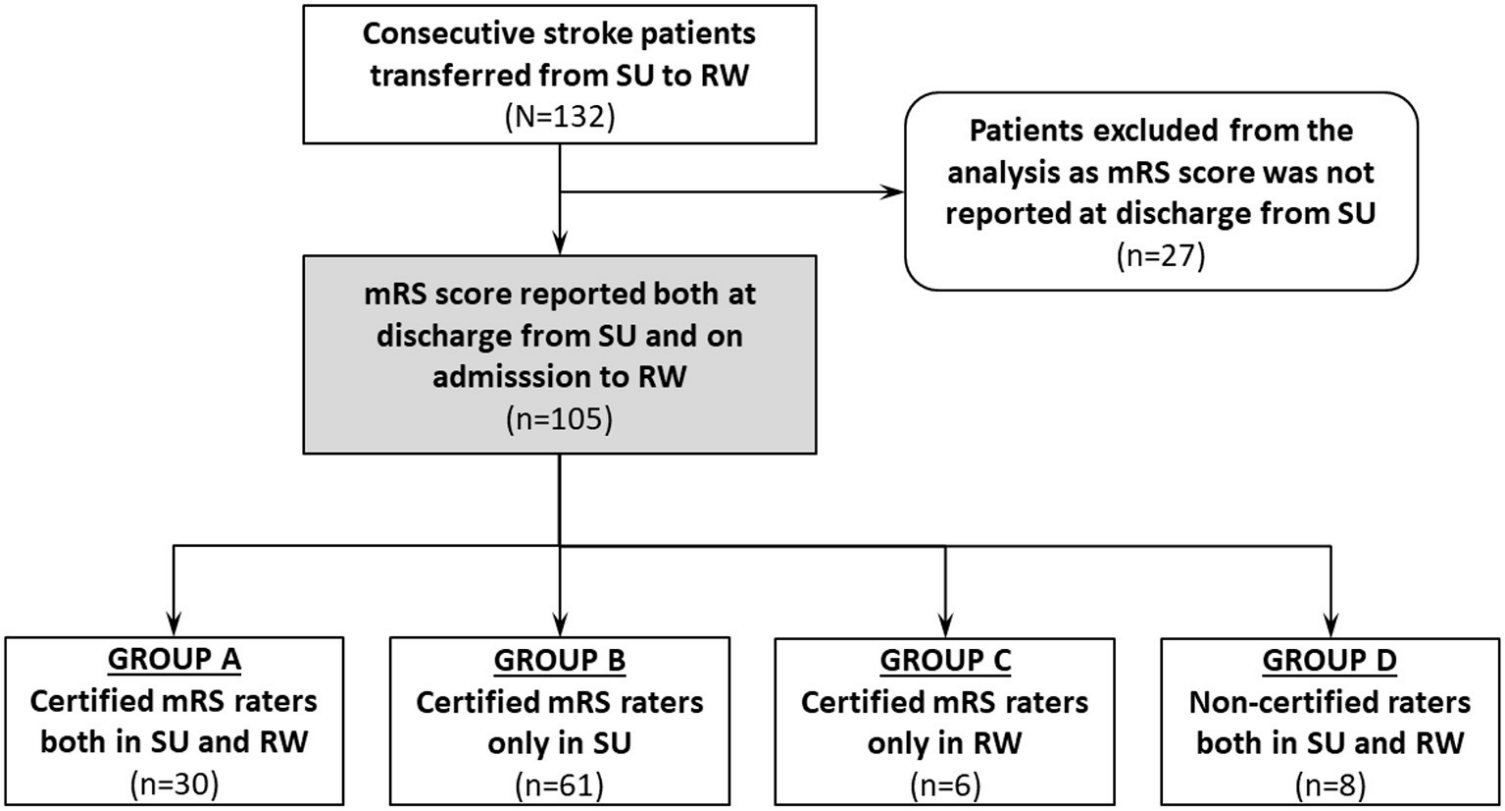
Study population. mRS, modified Rankin scale; RW, rehabilitation ward; SU, stroke unit.

Categorical variables were presented as a number of valid observations and proportions calculated with exclusion of unknown values from the denominator. Continuous variables were presented as a median with interquartile range (1^st^ quartile to 3^rd^ quartile, Q1–Q3) due to non-normal distribution. Consistency between the raters from SU and the raters from RW was expressed using Cohen's kappa. Considering potential differences in competence, we also planned to calculate the agreement in the following subgroups: (A) certified mRS rater in SU and certified mRS rater in RW; (B) certified mRS rater in SU and non-certified mRS rater in RW; (C) non-certified mRS rater in SU and certified mRS rater in RW; (D) non-certified mRS rater in SU and non-certified mRS rater in RW (Figure 1).

We calculated the proportions of patients who on admission to the RW received mRS score that was either identical, higher or lower than the score given at the discharge from SU. Then we compared the proportions of higher mRS scores at the discharge from SU and on admission to RW. Additional sensitivity analysis was conducted to compare the patients included in the main analysis and the patients excluded due to lack of double mRS assessment made by both SU and RW physicians.

Calculations were carried out using STATISTICA 13.3 software package (TIBCO Software Inc., USA). For comparisons Chi square test, two-tailed Fisher's exact and Mann-Whitney U test were used, as appropriate. *P* values of <0.05 were considered statistically significant.

## Results

During the 24-month study period a total of 132 acute stroke patients were transferred directly from SU to RW, of whom 105 (79.5%) had the mRS score reported both at discharge from SU and on admission to RW. The remaining 27 patients had no information about the mRS score at discharge from SU and therefore were excluded from the main analysis (Figure 1). Compared to the analyzed cohort, excluded patients were significantly more often male (77.8 vs. 49.5%, *p* = 0.008) and less often discharged from SU by a certified mRS rater (70.4 vs. 86.7%, *p* = 0.043) (Table 1).

**Table 1:** Basic characteristics of the analyzed cohort and patients excluded due to lack of double mRS assessment.

	**Patients with double mRS assessment (*n =* 105)**	**Patients without mRS assessment at discharge from the stroke unit (*n =* 27)**	***P* value**
Age (years), median (Q1–Q3)	73 (64–79)	70 (61–79)	0.456
Male sex, *n* (%)	52 (49.5)	21 (77.8)	0.008
Ischemic stroke, n (%)	94 (89.5)	24 (88.9)	1.00
Pre-stroke mRS 0–2, *n* (%)^*^	81 (81.0)	24 (96.0)	0.067
Reperfusion therapy, *n* (%)		
Intravenous rtPA	34 (32.4)	10 (37.0)	0.647
Mechanical thrombectomy	13 (12.4)	3 (11.1)	1.00
NIHSS on admission to stroke unit, median (Q1–Q3)	8 (4.5–15)	8 (5–14)	1.00
NIHSS at discharge from stroke unit, median (Q1–Q3)	5 (3–10.5)	7 (4-10)	0.311
Duration of stroke unit stay (days), median (Q1–Q3)	15 (13–21)	14 (11–20)	0.209
Certified mRS rater in stroke unit, *n* (%)	91 (86.7)	19 (70.4)	0.043
Certified mRS rater in rehabilitation ward, *n* (%)	36 (34.3)	14 (51.9)	0.120

mRS, modified Rankin Scale; ^*^n = 125, patients with reported pre-stroke mRS; Q1, first quartile; Q3, third quartile; rtPA, thrombolysis; NIHSS, National Institutes of Health Stroke Scale.

Patients from the analyzed cohort had a median age of 73 years, were in 89.5% independent (mRS 0–2) before stroke and were transferred to RW with median of 5 points at National Institutes of Health Stroke Scale (NIHSS) after a median of 15 days of stay in SU (Table 1). The proportion of patients assessed by a certified mRS rater was significantly higher in SU (86.7 vs. 34.3%, *p* <0.001).

Regardless of raters' certification status, the neurologists discharging from SU and the physicians admitting to RW reported identical mRS scores in 70.5% with Cohen's kappa 0.55 (Table 2). Similar findings were observed in subgroup A (*n* = 30; 70.0% of identical scores, kappa 0.57) and subgroup B (*n* = 61, 73.8% of identical scores, kappa 0.58). PRM physicians more often reported higher scores than SU physicians overall (20.0 vs. 9.5%, *p* = 0.050) and in subgroup A (26.7 vs. 3.3%, *p* = 0.026), however such difference was not observed in subgroup B (14.8 vs. 11.5%, *p* = 0.592). The number of cases in subgroups C (*n* = 6) and subgroup D (*n* = 8) was too low for detailed reporting and further statistical analysis.

**Table 2:** Interrater consistency in mRS scoring in stroke unit and rehabilitation ward.

	**All raters (*n =* 105)**	**GROUP A certified raters both in the stroke unit and the rehabilitation ward (*n =* 30)**	**GROUP B certified raters in the stroke unit and non-certified raters in the rehabilitation ward (*n =* 61)**
Identical mRS scores, *n* (%)	74 (70.5)	21 (70.0)	45 (73.8)
Higher mRS on admission to rehabilitation ward, *n* (%)	21 (20.0)	8 (26.7)	9 (14.8)
Lower mRS on admission to rehabilitation ward, *n* (%)	10 (9.5)	1 (3.3)	7 (11.5)
Cohen's kappa	0.55	0.57	0.58

mRS, modified Rankin Scale.

A matrix of mRS scores for subgroups A and B are presented in Table 3 and a matrix of mRS scores incorporating all 132 patients (including cases with not recorded mRS score at discharge from SU) are presented in Table 4.

**Table 3:** Cross tabulation of mRS scores for subgroups of patients depending on the certification status of raters (subgroups A and B).

	**Assessment at discharge from the stroke unit (certified rater)**					
		**mRS 1**	**mRS 2**	**mRS 3**	**mRS 4**	**mRS 5**
Group A assessment on admission to the rehabilitation ward (certified rater)	mRS 1		0% (0)			
	mRS 2		80% (4)	8% (1)		
	mRS 3		20% (1)	62% (8)	0% (0)	
	mRS 4			31% (4)	73% (8)	
	mRS 5				27% (3)	100% (2)
	All		5	13	11	2
	**Assessment at discharge from the stroke unit (certified rater)**					
		**mRS 1**	**mRS 2**	**mRS 3**	**mRS 4**	**mRS 5**
Group B assessment on admission to the rehabilitation ward (non-certified rater)	mRS 1	100% (1)	13% (1)			
	mRS 2		75% (6)	14% (3)		
	mRS 3		13% (1)	62% (13)	10% (3)	
	mRS 4			24% (5)	80% (24)	
	mRS 5				10% (3)	100% (0)
	All	1	8	21	30	0

mRS, modified Rankin Scale.

**Table 4:** Cross tabulation of mRS scores for all analyzed patients.

	**Discharge rating from the stroke unit**						
		**mRS 1**	**mRS 2**	**mRS 3**	**mRS 4**	**mRS 5**	**Not reported**
Admission rating to the rehabilitation ward	mRS 1	100% (1)	8% (1)				
	mRS 2		77% (10)	13% (5)			1
	mRS 3		15% (2)	60% (24)	8% (4)		6
	mRS 4			28% (11)	76% (37)		16
	mRS 5				16% (8)	100% (2)	4
	All	1	13	40	49	2	27

mRS, modified Rankin Scale.

## Discussion

The first version of scale was developed by John Rankin more than 60 years ago and consisted of 5 briefly described states (1 to 5) with no clear criteria for distinguishing between particular levels of disability (11). The scale was modified in the year 1988 by adding grades 0 and 6, which made the scale capable of capturing the whole spectrum of functional states (from no symptoms to death) but still burdened with significant interobserver variability (3, 7, 13, 14).

The first attempts to improve the reliability of mRS assessment included development of simple structured interview and subsequently the development of smRSq (8–10). The smRSq questionnaire includes a few pre-defined yes-or-no questions which helps to distinguish between different levels of post-stroke disability (8–10). The smRSq shows adequate agreement with the standard mRS and it can be useful in standard face-to-face interviews, telephone interviews or in creating online forms. Its reliability is superior to the standard mRS, but still not optimal (10, 11, 15, 16). Another attempt was the introduction of certification video-based training provided by Glasgow University (*trainingcampus.net*). Unfortunately, the training is available only in English and certification is not free from charge.

Suboptimal consistency of assessment driven by general rules biased by physicians' intuition led to development Rankin Focused Assessment (RFA). The RFA is an independent tool with a checklist that incorporates a detailed structured interview in line with mRS (4). Such form urges the rater to ask all pivotal questions and increases the reliability up to 93% (4, 16). Other proposed approaches to optimize functional assessment after stroke include miFUNCTION scale and utility-weighted mRS (UW-mRS) (16–21).

Unlike previous analyzes addressing the issue of interobserver variability of mRS scoring, our study provides real-life data about performance of physicians using the scale for the purpose of their everyday practice in the early post-acute phase of stroke. All raters were experienced in treating patients after stroke but they represented two different types of clinical background (SU and RW). Their mRS assessments were made with no pressure on precision, that may be exerted by awareness of being a clinical trial investigator or a participant in a validation project. The physicians could use any tool to capture the mRS score. However, knowing the clinical routine in both departments, it may be assumed that they used either smRSq or original mRS, not the RFA. The observed overall consistency of 70.5% with Cohen's kappa 0.55 shown in our study is similar to what has been reported for the smRSq in validation studies (8–12).

Interestingly, the nominal raters' competence understood as obtaining the mRS certificate for the purpose of clinical trials within the few preceding years did not seem to directly improve consistency. This may indicate that the added value of formal training program requires regular retraining or that the precision of certified raters is simply lower in the setting of everyday practice.

We found that the physicians admitting patients to RW had a strong tendency to rate disability higher than the neurologists discharging patients from SU. Noteworthy, this phenomenon was restricted to PRM physicians who completed the certification program. One may hypothesize that SU neurologist (i) tends to concentrate on the disability attributed only to the residual neurological deficits, (ii) only approximates how the patient could handle activities of daily living by observing him in an artificial SU environment and (iii) prefers to see the outcome of SU stay in a positive way. As a consequence, the SU neurologist may involuntarily underestimate the level of disability at discharge from SU.

On the other hand, PRM physician (i) may tend to perceive the patient's functional status in a more complex way including both activity and participation, (ii) is able to see the patient confronting more complex environment and his actual attempts to perform some activities of daily living in RW, and (iii) see the potential for measurable improvement during RW stay. As a consequence, the PRM physician may involuntarily overestimate the level of disability on admission to RW.

This abovementioned one-way skew disappeared when the assessment on admission to RW was done by a non-certified physician. In this subgroup (B) PRM physicians scored disability either higher or lower than the SU neurologists without any pattern. It may indicate that the formal training in mRS equalizes the competence and therefore reveals the background-related cognitive systematic error dependent on the rater background.

### Strengths and limitations

The most important strength of the presented study is the fact that it reflects real-life everyday practice. It is important to note that the moderate inconsistency indicates that the mRS reported on admission to RW is not a simple copy of what has been stated in the discharge note from SU. mRS is used as an outcome measure for both acute stroke studies and rehabilitation studies (15, 17). This is the first study that directly compares the mRS assessments between SU and PRM physicians and generates several hypotheses, which may deserve to be addressed in the future.

The retrospective design allowed to collect data unbiased by the awareness of raters that their performance will be externally evaluated or that their imprecise scoring may affect results of an important clinical trial. On the other hand, such method made impossible to decide which mRS assessment is correct in case of disagreement. It should also be noted that RW physicians were aware of the mRS scores given at the discharge from the SU. However, the level of discrepancy may indicate that both assessments were independent.

Transferring post-stroke patients from SU to RW is a common practice in stroke care (22, 23), but our findings refer directly to the subset of patients who require intensive neurological hospital rehabilitation and seem capable of benefiting from RW activities. In our cohort there were almost no patients without or with mild post-stroke disability (only one patient with mRS 1 and no cases with mRS 0) and almost no patients with severe disability (only two cases with mRS 5). Noteworthy, in Poland difficult access to reimbursed outpatient rehabilitation may lead to a higher proportion of patients with mRS 2 stayed in RW than in other health care systems.

The exclusion of a small subgroup of patients due to lack of mRS score stated directly at the time of discharge from SU was unlikely to introduce significant bias. The study accounted for all four combinations of certified and non-certified mRS raters. However, the number of observations was sufficient to perform statistical analysis only in two most important combinations (A and B).

## Conclusions

Our findings provide important reassurance that the reliability of mRS assessment made in everyday clinical practice of SU and RW is modest as in the validation studies. Therefore, it may be considered sufficient for the purpose of observational studies or stroke registries.

The tendency among neurologists to underrate disability at discharge from SU or the tendency among PRM physicians to overrate disability on admission to RW is a new observation that deserves further studies. Such studies should include additional reference mRS assessment to identify which physician scores correctly.

It seems reasonable to put additional efforts in improvement the reliability of mRS assessment either by incorporating repeated mRS training in the curriculum of all physicians involved in stroke care or by complementing of using specially designed mRS forms such as the RFA which navigates raters through the pivotal points of the interview.

## Data availability statement

The raw data supporting the conclusions of this article will be made available by the authors, without undue reservation.

## Ethics statement

Ethical review and approval was not required for the study on human participants in accordance with the local legislation and institutional requirements. Written informed consent for participation was not required for this study in accordance with the national legislation and the institutional requirements.

## Author contributions

NP conceived and designed the study, collected data, performed statistical analysis, interpreted results, drafted the manuscript, and provided approval of the final version for publication. IK-J, IS-D, and MN interpreted results, revised the manuscript for important intellectual content, and provided approval of the final version for publication. MK conceived and designed the study, performed statistical analysis, interpreted results, revised the manuscript for important intellectual content, and provided approval of the final version for publication. All authors contributed to the article and approved the submitted version.
